# Supplementary material for: Ni Nanoparticles on the Reduced Graphene Oxide Surface Synthesized in Supercritical Isopropanol
Source: Nanomaterials (Basel). 2023 Nov 9;13(22):2923. doi: 10.3390/nano13222923 (PMC10674343; doi:10.3390/nano13222923)
Supplement: Supplementary file 1 [file nanomaterials-13-02923-s001.zip › nanomaterials-2699026-supplementary.pdf]

Supplementary Information

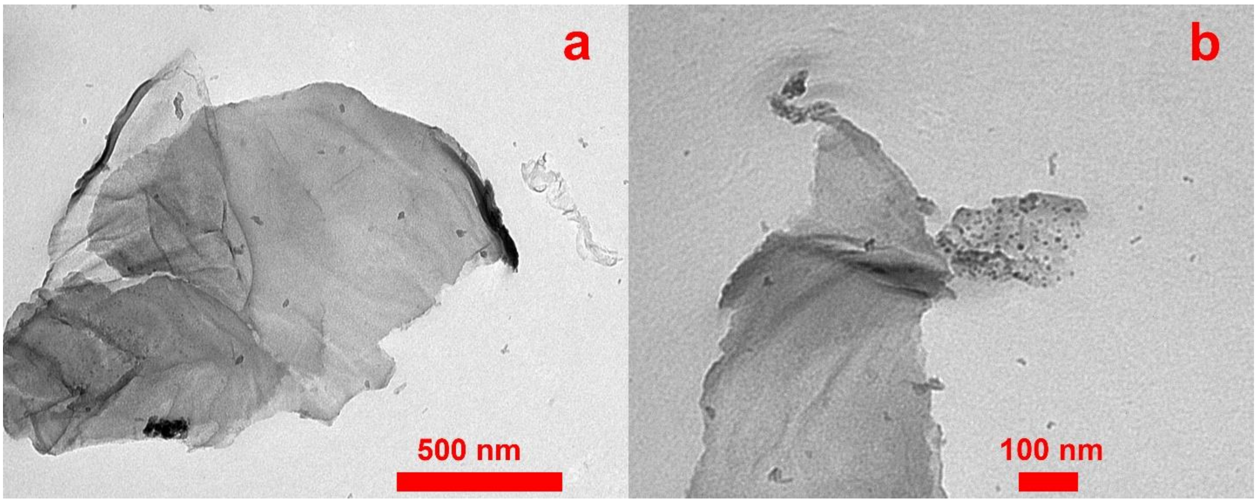

Figure S1. TEM of GO/ Ni^{2+} after thermal reduction without SCI treatment
